# Impact of different production systems on the slaughter performance and meat quality of gayal (*Bos frontalis*)

**DOI:** 10.3389/fvets.2025.1538622

**Published:** 2025-10-08

**Authors:** Li Zhi, Qing Li, Zibei Wang, Meng Zhang, Shichun He, Hao Zhang, Sifan Dai, Lijuan Zhang, Shusheng Zhao, Feng Jiang, Lixing Wang, Sheng He, Dingfu Kang, Chengming Duan, Maosheng Yang, Huaming Mao

**Affiliations:** ^1^Key Laboratory of Animal Nutrition and Feed Science of Yunnan Province, Yunnan Agricultural University, Kunming, China; ^2^Faculty of Animal Science, Xichang University, Xichang, China; ^3^Animal Husbandry Station of Yunnan Province, Kunming, China; ^4^Animal Husbandry Technology Extension Station of Nujiang Lisu Autonomous Prefecture, Lushui, China; ^5^Yunnan Tengsen Investment Group Limited, Tengchong, China; ^6^Livestock Work Station of Tengchong City, Tengchong, China

**Keywords:** altitude, different production systems, gayal, meat quality, slaughter performance

## Abstract

**Objective:**

The aim of this study was to evaluate the influence of different production systems, including low-altitude indoor feeding, high-altitude indoor feeding, and high-altitude grazing, on the slaughter performance and meat quality of gayals.

**Methods:**

We slaughtered 15 male gayals (five from each of three feedlots) that were randomly selected, with similar body weights within each feedlot. The gayals were raised under different production systems: indoor feeding at 1,325 m (TC, Tengchong feedlot; *n* = 5), indoor feeding at 2,240 m (FHS, Fenghuangshan feedlot; *n* = 5), and grazing at an altitude of 2,600 m (JMD, Jiumudang alpine pasture; *n* = 5). Gayals were slaughtered to evaluate slaughter performance, and the left longissimus dorsi (LD) and biceps femoris (BF) muscles were collected for meat quality analyses (pH_45min_, $L_{45\text{min}}^{*}$, ${\text{a}}_{45\text{min}}^{*}$, ${\text{b}}_{45\text{min}}^{*}$, muscle fiber diameter, water loss rate, shear force), chemical composition (moisture, crude protein, ether extract, cholesterol), amino acid composition, and fatty acid profile.

**Results:**

The results indicated that compared with the other two groups, the TC group exhibited significantly greater slaughter performance, including dressing percentage, lean meat percentage, and meat: bone ratio (*p* < 0.05). The TC group also showed higher levels of monounsaturated fatty acids (MUFA; especially C16:1 and C18:1 c9), essential amino acids (EAA), n-6:n-3 polyunsaturated fatty acids (PUFA) ratio, and thrombogenic index (TI), but lower cholesterol content (*p* < 0.05). In contrast, compared with the TC group, the JMD group had higher contents of PUFAs (including C18:3n3, C20:5n3, C22:5n3, and C22:6n3), a lower n-6:n-3 PUFA ratio, and a lower TI, with the TI decreased by 29.35% in LD muscle (*p* < 0.05). Additionally, the JMD group had higher contents of sweet amino acids in the LD muscle and umami amino acids in the BF muscle (*p* < 0.05). The slaughter performance and meat quality of the FHS group were intermediate between the TC and JMD groups, with closer alignment to the JMD group.

**Conclusion:**

Low-altitude indoor-fed gayals demonstrated superior slaughter performance and higher beef yield, whereas high-altitude grazed gayals produced beef with a more favorable fatty acid profile and enhanced flavor-related amino acids, despite exhibiting lower productivity. High-altitude indoor-fed gayals exhibited slaughter performance and meat quality indicators that were intermediate between the two systems.

## 1 Introduction

Gayal (*Bos frontalis*) is a distinctive bovid species characterized by its unique semi-wild and semi-domesticated attributes (1). The gayal diverged from the gaur approximately 994,000 years ago, forming a separate subspecies or even species, rather than a domestic form of gaur, and is likely domesticated from an extinct subspecies or species (2). Therefore, the gayal holds a unique and critical branch position in the evolution of bovine species. The gayal mainly inhabits the hill forest areas of China, India, Bangladesh, Myanmar, and Bhutan, and plays a significant role in promoting forest conservation and maintaining ecological balance (3).

In China, gayals are traditionally grazed in the alpine valleys and subtropical rainforests of the Dulong and Nujiang River basins, located in the high-altitude regions of Yunnan Province (4). In 2014, the gayal was officially recognized and integrated into the Nujiang Lisu Autonomous Prefecture of China's Records of Specialty Livestock and Poultry Resources. Nutritional analysis of gayal meat reveals a high protein content and low fat percentage, complemented by the presence of beneficial amino acids and fatty acids (5). Moreover, gayal meat possesses favorable meat quality traits, including a smaller muscle fiber diameter and increased tenderness (6). Consumer demand for this high-quality beef is increasing steadily. However, the current population of gayals in China is < 4,000 individuals, resulting in gayal beef commanding a market price nearly twice that of other beef types. As a consequence, gayal beef production plays a significant role in the local agricultural economy.

Different production systems have direct effects on slaughter performance and meat quality (7). Previous studies have demonstrated that grain-fed bison (8) and grain-fed yaks (9) exhibited significantly superior slaughter performance, including higher carcass weight and dressing percentage, compared to their grass-fed counterparts, and, in terms of meat quality and nutritional value, grain-fed beef had lower moisture levels, higher intramuscular fat (IMF) content, and significantly improved tenderness, whereas grass-fed beef had a more favorable fatty acid profile. Specifically, grass-fed beef had lower levels of cholesterol-raising saturated fatty acids (C12:0–C16:0) and higher total n-3 polyunsaturated fatty acids (PUFA) content, as well as increased levels of long-chain n-3 PUFAs such as C20:5n3, C22:5n3, and C22:6n3, therefore, conferring greater health benefits and providing enhanced protection for the cardiovascular system (10). Furthermore, beef from different altitudes exhibited significant differences in meat quality after slaughter. As altitude increased, the a^*^ value, pH, and shear force of beef significantly increased, whereas the b^*^ value, L^*^ value, and water-holding capacity significantly decreased (11). Additionally, previous reports indicated that yaks subjected to low-altitude indoor feeding exhibited higher dressing percentages, lean meat percentages, and overall edible meat quality compared with yaks grazing at high altitudes (12).

In China, due to the low efficiency of beef production under the high-altitude grazing system, some farmers have relocated gayals to other feedlots for indoor feeding, which are located at different altitudes. Nonetheless, to date, no systematic evaluations of their slaughter performance and meat quality under different production systems have been conducted. Therefore, the current study was conducted to evaluate the slaughter performance and meat quality of gayals under different production systems, including low-altitude indoor feeding, high-altitude indoor feeding, and high-altitude grazing conditions. The objective was to provide a comprehensive reference for the efficient and sustainable farming of gayals.

## 2 Materials and methods

### 2.1 Ethics statement

This research was approved by the Laboratory Animal Management and Use Committee of Yunnan Agricultural University (Approval No. YNAU 20180020).

### 2.2 Experimental animals

The study was conducted by randomly selecting five male gayals (~3 years old) for slaughter from each of the three different feedlots, totaling 15 male gayals. The three feedlots employed distinct production systems. In the low-altitude indoor feeding system (TC group), five healthy gayals were randomly selected based on similar body weight from the Tengchong feedlot in Qushi Town, Yunnan Province, China (altitude 1,325 m). These gayals had grazed at altitudes ranging from 2,500 m to 3,200 m until the age of 2 years before being transferred to the feedlot, with the fattening period lasting for 317 days (initial weight 261.1 ± 30.2 kg). For the high-altitude indoor feeding system (FHS group), five healthy gayals with matched body weights were randomly selected from the Fenghuangshan feedlot located in Lushui City, Yunnan Province, China (altitude 2,240 m). These gayals (initial weight 294.4 ± 66.4 kg) were subjected to a fattening period of only 47 days at the feedlot, having previously grazed at an altitude of 2,600 m before entering the fattening facility. Both the TC and FHS groups were provided with a corn silage-based diet. In the high-altitude grazing system (JMD group), five healthy gayals were selected from the Jiumudang alpine pasture in Gongshan County, Yunnan Province, China (altitude 2,600 m) to ensure similar initial body weights. These gayals were grazed year-round in alpine valleys where the plants primarily consist of alpine arrow bamboo, reeds, artemisia, and weeds. The ingredients and chemical compositions of the diets for the three experimental groups are shown in Table 1.

**Table 1:** The ingredient and chemical compositions in the diets (dry-matter basis).

**Items**	**Indoor supplementary feeding diet**		**Grazing alpine valleys^1^**
	**TC (*n* = 5)**	**FHS (*n* = 5)**	**JMD (*n* = 5)**
**Ingredient**			
Corn silage	59.5	58.3	
Concentrate	37.3	38.2	
Corn grain	3.2	–	
Wheat bran	–	3.5	
Total	100.0	100.0	
**Nutrient levels**			
NEg3 × (Mcal/kg)	0.96	0.93	0.46
CP (%)	12.12	11.86	7.55
NDF (%)	43.54	42.42	69.16
ADF (%)	24.96	23.61	45.96
Ca (%)	0.82	0.87	0.12
P (%)	0.45	0.52	0.19

^1^The plants primarily consist of alpine arrow bamboo, reeds, artemisia, and weeds.

TC, indoor feeding at 1,325 m; FHS, indoor feeding at 2,240 m; JMD, grazing at 2,600 m; NEg3 × , net energy for gain; CP, crude protein; NDF, neutral detergent fiber; ADF, acid detergent fiber.

The nutritional value of corn silage in the TC group were NEg3 × 0.79 Mcal/kg, CP 8.76%, NDF 52.73%, ADF 33.63%, Ca 0.41%, P 0.19%; The nutritional value of corn silage in the FHS group were NEg3 × 0.78 Mcal/kg, CP 7.75%, NDF 49.69%, ADF 31.31%, Ca 0.46%, P 0.25%; composition of the raw materials for concentrate supplementation were corn 61%, soybean meal 9.3%, rapeseed meal 7.0%, bran 15%, calcium hydrogen phosphate 2%, calcium carbonate 2.3%, sodium chloride 1%, sodium bicarbonate 0.4%, premix 2%. The doses per kilogram of premix were as follows: 5,000 IU vitamin A, 6,000 IU vitamin D, 5,000 mg vitamin E, 15 mg Cu, 100 mg Fe, 60 mg Zn, 100 mg Mn, 0.7 mg Se, 2.0 mg I, and 0.90 mg Co.

### 2.3 Slaughter performance measurements

All slaughter procedures followed the China National Operation Procedure of Cattle Slaughtering Standards (GB/T 19477-2018). Animals were fasted for 24 h and deprived of water for 8 h before slaughter. They were stunned after weighing and then exsanguinated, with the viscera, digestive system, head, skin, and hooves removed. The meat, bone, viscera and adipose tissue were also weighed in order to evaluate the slaughter performance and carcass characteristics. Dressing percentage was calculated as carcass weight divided by slaughter weight. Lean meat percentage was determined by dividing the lean meat weight by the carcass weight. Meat–bone ratio was calculated as a ratio of lean meat weight to bone weight. The backfat thickness of the left carcasses was measured over the deepest part of the loin-eye muscle (13), specifically, after the entire carcass was split into left and right halves, the backfat thickness was measured at the cross-sectional area between the 12th and 13th ribs on the left side of the carcass using a digital vernier caliper, with the measurement taken perpendicular to the muscle surface. Subsequently, the color of fat was evaluated using a color chart (1 = pure white, 8 = yellow), and the fat color was categorized into eight levels based on color depth (14), with a larger number indicating a yellower color. The cross-sectional area of the longissimus dorsi (LD) muscle between the 12th and 13th ribs was outlined on sulfuric acid drawing paper, and then the loin eye area was calculated using a digital planimeter (LK-300, Kezhe Biotech Co., Ltd., Shanghai, China) (15). This involved placing the sulfuric acid drawing paper on the LK-300 measuring platform, scanning the contour using an automatic sensor, and automatically calculating the enclosed area as the loin eye area.

After the animals were slaughtered, the carcasses were segmented according to the China National Beef Carcass and Cuts Standards (GB/T 27643-2011). Subsequently, samples of the LD muscle at the 12th and 13th ribs and the biceps femoris muscle (BF, muscle from the lateral hind legs located along the femoral margin of the semitendinosus muscle) were taken from the left half of each carcass and divided into two portions. One portion of the LD and BF muscles was used for on-site analysis of meat quality traits, including pH (45 min), L^*^ (45 min), a^*^ (45 min), b^*^ (45 min), muscle fiber diameter, and water loss rate. The other portion was immediately sealed in sterile vacuum bags and stored at −20 °C for subsequent analysis of shear force, chemical composition (moisture, crude protein, ether extract, cholesterol), amino acid composition, and fatty acid profile.

### 2.4 Meat quality and chemical composition measurements

The pH value was measured by directly inserting an electrode 45 min after slaughter (1.5 cm deep into the muscle). The pH meter (BJ-260, Leici Co., Ltd., Shanghai, China) had been previously calibrated with pH 4.0 and pH 7.0 buffers. Muscle fiber diameter was determined following established methods from previous studies (16). The samples were placed in a constant temperature water bath and reaching a center temperature of 70 °C for 20 min, the meat was cooled to room temperature. Shear force was measured using a texture analyser (Model C-LM 3, Harbin Instruments Inc., China) on three cooked meat cores per sample (cylindrical, diameter 1.27 cm, length 3 cm) (17), with the mean value recorded after repeating the process three times. Meat colorimetric traits (L^*^ value, a^*^ value, and b^*^ value) were measured at 45 min postmortem using a chromometer (CR-300, Minolta Co., Ltd., Osaka, Japan) at three randomly selected locations, and the average values were calculated (18). The chromameter was calibrated with a standardized white plate before each measurement. Water loss rate was measured using a texture analyser (Stable Micro Systems Inc., Godalming, UK). The middle part of the left LD and BF muscles were trimmed to 30 mm × 35 mm × 2 mm, weighed. The meat samples were placed between 20 pieces of filter paper and pressed at 35 kg for 300 s. Then, the samples were weighed again. The water loss rate was calculated in accordance with the methodology outlined by Ge et al. (19). The moisture, crude protein and ether extract of the muscle were determined using the AOAC method (20). According to the China National Standards for Determination of Cholesterol in Foods (GB 5009.128-2016), HPLC was used to determine cholesterol levels and the external standard method was used for quantification. The samples were homogenized and 1 g of samples was placed in a tube, mixed with 5 ml (60% KON: ethanol = 1:2) solution, and bathed in water at 70 °C until no precipitation occurred. After cooling, 10 ml (diethyl ether: petroleum ether = 1:1) solution was added, shaken, and centrifuged (9,000 rpm, 5 min). The waste liquid in the lower layer was discarded. The remaining solution was nitrogen-blown until dry. After drying, 5 ml anhydrous ethanol was added. The sample was filtered through a 0.45 μm membrane into a sample bottle for computerized testing.

### 2.5 Fatty acid measurements

The determination of fatty acid levels was optimized based on the method of the China National Determination of Fatty Acids in Foods Standards (GB 5009.168-2016). After homogenizing the muscle, 30 mg of sample was transferred to a 50 ml EP tube and mixed with 500 μl of extraction solution (isopropanol: hexane = 2:3). The mixture was vortexed for 30 s and ground for 4 min at 40 Hz using a grinding instrument. After that, it was ultrasound-treated in an ice water bath for 5 min, repeated thrice. The samples were centrifuged at 12,000 rpm and 4 °C for 15 min, and the supernatant was then transferred to another EP tube. The combined supernatant was concentrated by nitrogen blowing, dried completely, and then treated with a methanol: trimethylsilyl diazomethane (1:2) solution was maintained at room temperature for 30 min before being blow-dried again with nitrogen. Finally, hexane was added to redissolve the sample before centrifuging at high speed and filtering through a membrane (0.45 μm) into the loading bottle. Fatty acid analysis was conducted using a gas chromatography-mass spectrometry (GC-MS) instrument (Trace 1310 ISQ, Thermo, USA). The atherogenic index (AI), thrombogenic index (TI) and hypocholesterolemic: hypercholesterolemic ratio (h:H) were calculated according to the following equations (7, 21):


(1) $$\begin{array}{l} \text{AI}=[\text{C12}:\text{0}+4\times \text{C14}:\text{0}+\text{C16}:\text{0}]/[\sum \text{MUFA}\\ \;+\sum \text{n}-\text{6}+\sum \text{n}-\text{3}] \end{array}$$

(2) $$\begin{array}{l} \text{TI}=[\text{C14}:\text{0}+\text{C16}:\text{0}+\text{C18}:\text{0}]/[0.5\times \sum \text{MUFA}+0.5\\ \;\times \sum \text{n}-\text{6}+3\times \sum \text{n}-\text{3}+\sum \text{n}-\text{3}/\sum \text{n}-\text{6}] \end{array}$$

(3) $$\begin{array}{l} \text{h}:\text{H}=[\text{C18}:\text{1n9c}+\sum \text{n}-\text{6}+\sum \text{n}-\text{3}]/[\text{C12}:\text{0}+\text{C14}:\text{0}\\ \;+\text{C16}:\text{0}] \end{array}$$

### 2.6 Amino acid measurements

Following the methods outlined in the China National Determination of Amino Acids in Foods Standards (GB 5009.124-2016), ion-exchange chromatography was used with post-column derivatization of ninhydrin to conduct the amino acid analysis. The samples were hydrolyzed with 10 ml of 6 mol/L HCl and three to four drops of phenol at 110 ± 1 °C for 22 h. The mixed amino acid standard solution and the sample solution were injected into the amino acid analyzer in equal volumes. The concentration of amino acid in the sample determination solution was calculated using the peak area of the external standard method.

### 2.7 Statistical analysis

All analyses were conducted using the General Linear Model (GLM) procedure in IBM SPSS Statistics (version 25.0). The effect of the three production systems on all variables was analyzed using a statistical linear model: *Y*_*ij*_ = μ + *M?* + *e*_*ij*_, where *Y*_*ij*_ is the observed value for the j-th animal within the i-th production system (i = TC, FHS, JMD), μ is the overall mean, *M*_*i*_ is the fixed effect of the i-th production system, and *e*_*ij*_ is the residual error for the j-th animal within the i-th production system. The level of significance between the groups was determined according to Tukey's HSD *post-hoc* test using *p* < 0.05 as the limit to identify significant differences. In addition, principal component analysis was conducted using Origin (version 9.8.0.200) to identify the components that absorb greater variability and separate the three production systems. Only the qualitative parameters that exhibited significant differences among the three groups were included in the analysis; these parameters refer to indicators of slaughter performance and meat quality of the LD muscle, such as dressing percentage, pH value, and amino acid composition.

## 3 Results

### 3.1 Slaughter performance

Data on slaughter performance are presented in Table 2. The dressing percentage, lean meat percentage, meat: bone ratio, and backfat thickness in the TC group were significantly higher than those in the FHS and JMD groups (*p* < 0.05). The loin eye area was also significantly larger in the TC group than in the JMD group (*p* < 0.05). The color of fat was lower in the FHS group than in the JMD group (*p* < 0.05). With regards to viscera tissue, compared to the TC group, the FHS group showed a higher lung weight proportion and a lower spleen weight proportion relative to slaughter weight (*p* < 0.05). The proportion of kidney weight relative to slaughter weight in the JMD group was significantly higher than that in the TC group (*p* < 0.05).

**Table 2:** Comparison of the differences in slaughter performance of gayals under different production systems.

**Items**	**Treatment**			**SEM**	***p*-value**
	**TC (*n* = 5)**	**FHS (*n* = 5)**	**JMD (*n* = 5)**		
**Carcass characteristics**					
Slaughter weight (kg)	338.93	323.91	382.89	17.50	0.387
Carcass weight (kg)	214.89	188.23	210.83	11.96	0.653
Dressing percentage (%)	63.11^a^	57.39^b^	54.97^b^	1.20	0.006
Lean meat weight (kg)	172.14	145.63	166.59	10.22	0.571
Lean meat percentage (%)	50.47^a^	44.32^b^	43.23^b^	1.15	0.009
Meat/bone	4.93^a^	3.71^b^	4.04^b^	0.21	0.027
Backfat thickness (mm)	24.20^a^	8.51^b^	5.80^b^	2.40	< 0.001
Loin eye area (cm^2^)	77.81^a^	61.07^ab^	58.16^b^	3.81	0.043
Color of fat	4.60^ab^	3.60^b^	5.30^a^	0.26	0.012
**Viscera tissue**					
Heart/slaughter weight (%)	0.42	0.49	0.46	0.02	0.447
Liver/slaughter weight (%)	1.22	1.23	1.07	0.06	0.539
Spleen/slaughter weight (%)	0.29^a^	0.17^b^	0.22^ab^	0.02	0.035
Lung/slaughter weight (%)	0.77^b^	1.12^a^	0.88^ab^	0.06	0.017
Kidney/slaughter weight (%)	0.16^b^	0.22^ab^	0.24^a^	0.01	0.020

SEM, standard error of the mean; TC, indoor feeding at 1,325 m; FHS, indoor feeding at 2,240 m; JMD, grazing at 2,600 m; dressing percentage was calculated as carcass weight divided by slaughter weight; lean meat percentage was determined by dividing the lean meat weight by the carcass weight; meat–bone ratio was calculated as a ratio of lean meat weight to bone weight.

^a, b^Differing superscript letters within the same rows indicate significant differences (p < 0.05).

### 3.2 Meat quality and chemical composition

The meat quality and chemical composition data are shown in Table 3. At 45 min postmortem, the pH values in the FHS and JMD groups were significantly higher than those in the TC group (*p* < 0.05). The LD muscle fiber diameter was larger in the TC and JMD groups compared to the FHS group (*p* < 0.05), and the BF muscle fiber diameter increased in the TC group than in the FHS and JMD groups (*p* < 0.05). Regarding color parameters, the BF muscle from the FHS group exhibited lower b^*^ values than those in the TC and JMD groups (*p* < 0.05). In terms of chemical composition, the crude protein content was higher in the LD muscle of the TC group compared to the FHS group (*p* < 0.05), whereas the highest crude protein content in the BF muscle was observed in the JMD group (*p* < 0.05). Additionally, it was observed that the ether extract content was highest in the TC group, but the cholesterol level was lowest among the three groups (*p* < 0.05).

**Table 3:** Comparison of the differences in meat quality and chemical composition of gayals under different production systems.

**Items**	**Longissimus dorsi**					**Biceps femoris**				
	**TC (*n* = 5)**	**FHS (*n* = 5)**	**JMD (*n* = 5)**	**SEM**	***p*-Value**	**TC (*n* = 5)**	**FHS (*n* = 5)**	**JMD (*n* = 5)**	**SEM**	***p*-Value**
**Meat quality**										
pH (45 min)	5.89^b^	6.26^a^	6.03^a^	0.08	0.035	6.14^b^	6.48^a^	6.40^a^	0.06	0.033
Muscle fiber diameter (μm)	32.32^a^	30.22^b^	31.99^a^	0.30	0.001	43.89^a^	33.30^b^	33.88^b^	1.31	< 0.001
Shear force (*N*)	47.97	43.46	47.44	0.96	0.103	53.44	47.77	49.29	1.52	0.311
Water loss rate (%)	25.10	26.17	27.56	1.29	0.065	18.04	19.82	22.18	0.79	0.615
L^*^ (45 min)	32.54	30.45	29.85	0.96	0.517	30.94	30.71	29.13	0.52	0.321
a^*^ (45 min)	8.48	9.13	9.55	0.66	0.823	11.45	11.62	11.94	0.27	0.778
b^*^ (45 min)	6.94	6.68	6.99	0.42	0.956	8.69^a^	7.09^b^	8.92^a^	0.38	0.048
**Chemical composition**										
Moisture (%)	75.66	78.22	78.32	0.60	0.110	79.98^a^	78.64^a^	76.93^b^	0.41	0.001
Crude protein (%)	23.57^a^	20.76^b^	22.1^ab^	0.35	0.002	19.68^c^	21.21^b^	22.45^a^	0.27	< 0.001
Ether extract (%)	1.81^a^	0.56^b^	0.50^b^	0.13	< 0.001	0.87^a^	0.56^b^	0.58^b^	0.06	0.035
Cholesterol (mg/100 g)	23.36^b^	32.3^a^	30.98^a^	1.21	< 0.001	25.16^b^	33.18^a^	34.22^a^	1.14	< 0.001

SEM, standard error of the mean; TC, indoor feeding at 1,325 m; FHS, indoor feeding at 2,240 m; JMD, grazing at 2,600 m; L^*^, lightness; a^*^, redness; b^*^, yellowness.

^a, b, c^Differing superscript letters within the same rows indicate significant differences (p < 0.05).

### 3.3 Fatty acid composition

The results regarding the differences in fatty acid composition among various production systems are presented as follows in Table 4. While the fatty acid profiles were generally similar between the two muscle types, some notable differences were observed. The LD muscle exhibited higher contents of C16:0 and C18:1 c9 across all three groups compared to the BF muscle. In contrast, the BF muscle showed a tendency toward higher levels of C18:2n6c, C20:4n6, total PUFA content, and the n-6:n-3 PUFA ratio and the h:H ratio.

**Table 4:** Comparison of the differences in fatty acid composition of gayals under different production systems (%).

**Items**	**Longissimus dorsi**					**Biceps femoris**				
	**TC (*n* = 5)**	**FHS (*n* = 5)**	**JMD (*n* = 5)**	**SEM**	***p*-Value**	**TC (*n* = 5)**	**FHS (*n* = 5)**	**JMD (*n* = 5)**	**SEM**	***p*-Value**
**SFA**										
C12:0	0.0047^b^	0.0311^a^	0.039^a^	0.00	0.003	0.0072^c^	0.0333^b^	0.0484^a^	0.00	< 0.001
C13:0	0.0026^b^	0.006^ab^	0.0087^a^	0.00	0.006	0.003^b^	0.0052^ab^	0.0092^a^	0.00	0.006
C14:0	0.99^a^	0.48^b^	0.41^b^	0.10	0.029	0.64	0.42	0.50	0.10	0.685
C15:0	0.20^b^	0.27^b^	0.44^a^	0.03	< 0.001	0.18^b^	0.25^b^	0.40^a^	0.03	0.001
C16:0	17.21	16.11	14.75	0.66	0.329	16.72	14.82	14.33	0.72	0.387
C17:0	0.60^b^	0.59^b^	0.83^a^	0.05	0.047	0.55^b^	0.50^b^	0.75^a^	0.05	0.046
C18:0	14.41^b^	17.29^ab^	19.42^a^	0.73	0.006	15.70	17.57	19.30	0.64	0.058
C20:0	0.11^c^	0.20^b^	0.25^a^	0.02	< 0.001	0.12^c^	0.18^b^	0.25^a^	0.02	< 0.001
C21:0	0.0069^c^	0.0347^b^	0.0525^a^	0.01	< 0.001	0.0071^c^	0.0353^b^	0.0539^a^	0.01	< 0.001
C22:0	0.0164^b^	0.0951^a^	0.1159^a^	0.01	< 0.001	0.0276^b^	0.0916^a^	0.1124^a^	0.01	< 0.001
C23:0	0.0191^c^	0.0511^b^	0.0724^a^	0.01	< 0.001	0.0222^c^	0.0505^b^	0.07^a^	0.01	< 0.001
C24:0	0.0206^c^	0.0394^b^	0.0532^a^	0.00	< 0.001	0.0249^c^	0.0384^b^	0.0529^a^	0.00	< 0.001
**MUFA**										
C14:1	0.30	0.18	0.16	0.03	0.105	0.13	0.20	0.18	0.02	0.374
C16:1	2.80^a^	1.36^b^	0.86^b^	0.26	0.001	1.94	1.47	0.86	0.20	0.079
C18:1 c9	33.57^a^	16.64^b^	13.5^b^	2.75	< 0.001	25.51^a^	15.96^b^	12.40^b^	2.17	0.023
C18:1 t11	5.94	16.47	12.87	2.31	0.170	0.28^b^	14.36^a^	14.09^a^	2.13	0.001
C18:1 c11	2.33^a^	1.48^b^	1.53^b^	0.14	0.010	2.48^a^	1.64^b^	1.54^b^	0.15	0.008
C20:1	0.19	0.15	0.14	0.01	0.279	0.18	0.11	0.16	0.01	0.114
**PUFA**										
C18:2n6t	0.0357^b^	0.099^a^	0.0961^a^	0.01	0.001	0.0529^b^	0.0749^ab^	0.0928^a^	0.01	0.009
C18:2n6c	8.72^b^	11.83^ab^	14.02^a^	0.89	0.036	14.64	13.98	14.29	0.97	0.968
C18:3n3	0.44^c^	2.52^b^	4.74^a^	0.49	< 0.001	0.66^c^	3.08^b^	4.96^a^	0.54	< 0.001
C18:3n6	0.07^b^	0.12^a^	0.10^ab^	0.01	0.005	0.10	0.12	0.09	0.01	0.076
C20:2	0.07^b^	0.25^a^	0.27^a^	0.03	< 0.001	0.16	0.2	0.16	0.01	0.271
C20:3n6	0.46^b^	0.83^a^	0.87^a^	0.07	0.009	0.85	0.75	0.82	0.04	0.602
C20:4n6	3.90^b^	7.35^a^	8.91^a^	0.69	0.001	7.58	8.35	9.39	0.50	0.364
C20:5n3	0.18^b^	1.59^a^	1.70^a^	0.19	< 0.001	0.38^b^	1.70^a^	1.64^a^	0.19	0.001
C22:4n6	0.34	0.40	0.35	0.02	0.618	0.57^a^	0.37^b^	0.31^b^	0.04	0.001
C22:5n6	0.08	0.11	0.10	0.01	0.116	0.15^a^	0.11^ab^	0.08^b^	0.01	0.049
C22:5n3	0.40^b^	1.97^a^	1.93^a^	0.21	< 0.001	0.72^b^	1.87^a^	1.72^a^	0.15	< 0.001
C22:6n3	0.07^b^	0.29^a^	0.28^a^	0.03	< 0.001	0.15^b^	0.33^a^	0.25^a^	0.02	0.001
∑SFA	33.59	35.2	36.45	1.09	0.597	34.01	34.03	35.90	0.99	0.704
∑MUFA	51.65^a^	37.3^b^	30.02^b^	2.73	< 0.001	39.97^a^	34.92^ab^	30.13^b^	1.66	0.039
∑PUFA	14.76^b^	27.49^a^	33.53^a^	2.43	< 0.001	26.03	31.05	33.97	1.89	0.234
PUFA/SFA	0.45^b^	0.81^a^	0.93^a^	0.07	0.007	0.78	0.92	0.98	0.07	0.488
MUFA/SFA	1.61^a^	1.07^ab^	0.83^b^	0.12	0.009	1.18^a^	1.03^ab^	0.85^b^	0.06	0.039
n-6/n-3	12.61^a^	3.24^b^	2.82^b^	1.27	< 0.001	13.11^a^	3.43^b^	2.92^b^	1.35	< 0.001
h/H	2.64	2.65	3.07	0.11	0.211	2.94	3.06	3.24	0.15	0.762
AI	0.32	0.28	0.26	0.02	0.389	0.29	0.25	0.26	0.02	0.731
TI	0.92^a^	0.71^ab^	0.65^b^	0.05	0.049	0.88	0.66	0.66	0.05	0.073

SEM, standard error of the mean;TC, indoor feeding at 1,325 m; FHS, indoor feeding at 2,240 m; JMD, grazing at 2,600 m; SFA, saturated fatty acids; MUFA, monounsaturated fatty acids; PUFA, polyunsaturated fatty acids; h, hypocholesterolemic; H, hypercholesterolemic; AI, atherogenic index; TI, thrombogenic index.

^a, b, c^Differing superscript letters within the same rows indicate significant differences (p < 0.05).

Significant differences in fatty acid composition were evident among the three production systems. In terms of saturated fatty acids (SFA), the JMD group exhibited higher SFA content in both muscles, with significantly elevated levels of C15:0, C17:0, C20:0, C21:0, C23:0, and C24:0 compared to the TC and FHS groups (*p* < 0.05). Only the C14:0 content in the LD muscle was higher in the TC group than in the other groups (*p* < 0.05). Regarding monounsaturated fatty acids (MUFA), the TC group showed significantly higher contents of C16:1, C18:1 c9, C18:1 c11, and total MUFA in the LD muscle, as well as elevated C18:1 c9 and C18:1 c11 levels in the BF muscle (*p* < 0.05). However, the C18:1 t11 content in the BF muscle was lowest in the TC group (*p* < 0.05). For PUFA, the FHS and JMD groups demonstrated significantly higher contents of multiple PUFAs, including C18:3n3, C20:5n3, C22:5n3, and C22:6n3 in both muscles compared to the TC group (*p* < 0.05). Additionally, in the LD muscle, C18:2n6t, C20:2, C20:3n6, C20:4n6, and total PUFA content were higher in the FHS and JMD groups than in the TC group (*p* < 0.05). The n-6:n-3 PUFA ratio was significantly lower in both muscles of the FHS and JMD groups compared to the TC group (*p* < 0.05). Furthermore, the TI in LD muscle was lower in the JMD group than in the TC group (*p* < 0.05).

### 3.4 Amino acid composition

Table 5 demonstrates that the muscle amino acid composition was significantly influenced by different production systems.

**Table 5:** Comparison of the differences in amino acid composition of gayals under different production systems (%).

**Items**	**Longissimus dorsi**					**Biceps femoris**				
	**TC (*n* = 5)**	**FHS (*n* = 5)**	**JMD (*n* = 5)**	**SEM**	***p*-Value**	**TC (*n* = 5)**	**FHS (*n* = 5)**	**JMD (*n* = 5)**	**SEM**	***p*-Value**
**Nonessential amino acids**										
Aspartic acid	9.95^a^	10.07^a^	9.73^b^	0.05	0.002	9.77^b^	10.06^a^	10.23^a^	0.06	< 0.001
Serine	3.68^b^	3.85^a^	3.76^ab^	0.03	0.032	3.75^b^	3.95^a^	3.96^a^	0.03	0.003
Glutamic acid	15.86	15.64	15.98	0.07	0.151	15.80	15.81	15.77	0.05	0.947
Glycine	4.51^b^	5.41^a^	4.53^b^	0.17	0.031	4.68	5.22	4.71	0.13	0.197
Alanine	6.01^b^	6.63^a^	6.70^a^	0.10	0.001	6.20	6.69	6.36	0.11	0.206
Cysteine	0.05^b^	0.27^a^	0.20^a^	0.03	0.006	0.17	0.22	0.27	0.03	0.605
Arginine	7.06	7.00	7.08	0.02	0.425	6.88	7.00	6.99	0.03	0.256
Proline	4.02	4.12	4.13	0.09	0.877	4.40^a^	4.16^ab^	3.89^b^	0.08	0.028
Tyrosine	4.04	3.93	4.06	0.03	0.114	3.97	3.93	3.95	0.03	0.843
NEAA	48.12^b^	49.91^a^	49.09^ab^	0.27	0.011	48.75^b^	50.04^a^	49.15^ab^	0.21	0.020
**Essential amino acids**										
Valine	5.32^a^	4.95^b^	4.71^c^	0.07	< 0.001	5.55^a^	4.88^b^	5.11^b^	0.09	0.001
Methionine	3.42^a^	3.44^a^	3.18^b^	0.04	0.002	3.41	3.50	3.44	0.02	0.218
Isoleucine	5.03^a^	4.69^b^	4.66^b^	0.06	0.001	4.87^a^	4.58^b^	4.83^a^	0.05	0.033
Leucine	8.59	8.34	8.60	0.11	0.565	8.43	8.37	8.40	0.04	0.844
Threonine	4.60	4.62	4.54	0.02	0.348	4.60	4.67	4.75	0.03	0.055
Phenylalanine	4.36	4.31	4.27	0.05	0.783	4.32	4.38	4.36	0.05	0.898
Histidine	3.64	3.27	3.52	0.09	0.236	3.69^a^	3.29^b^	3.48^ab^	0.07	0.041
Lysine	9.87^b^	9.47^b^	10.36^a^	0.11	< 0.001	9.50	9.27	9.50	0.05	0.102
EAA	51.88^a^	50.09^b^	50.91^ab^	0.27	0.011	51.25^a^	49.96^b^	50.85^ab^	0.21	0.020
**Flavor amino acids**										
Umami amino acids	25.81	25.70	25.71	0.07	0.800	25.57^b^	25.87^ab^	26.00^a^	0.08	0.047
Bitter amino acids	41.46^a^	39.93^b^	40.07^b^	0.23	0.002	41.12^a^	39.94^b^	40.55^ab^	0.18	0.015
Sweet amino acids	32.68^b^	34.1^a^	34.02^a^	0.25	0.015	33.13	33.97	33.18	0.19	0.121

SEM, standard error of the mean; TC, indoor feeding at 1,325 m; FHS, indoor feeding at 2,240 m; JMD, grazing at 2,600 m; EAA, essential amino acids; NEAA, nonessential amino acids; Umami amino acids: aspartic acid, glutamic acid; bitter amino acids: valine, methionine, isoleucine, leucine, tyrosine, phenylalanine, histidine, argnine; Sweet amino acids: glycine, serine, threonine, alanine, proline, lysine.

^a, b, c^Differing superscript letters within the same rows indicate significant differences (p < 0.05).

Regarding nonessential amino acids (NEAA), the total NEAA content in both the LD and BF muscles was higher in the FHS group than in the TC group (*p* < 0.05). In the LD muscle, the content of aspartic acid was significantly higher in the TC and FHS groups than in the JMD group (*p* < 0.05), whereas serine and glycine were lower in the TC group than in the FHS group (*p* < 0.05). Additionally, alanine and cysteine contents were lowest in the TC group (*p* < 0.05). In the BF muscle, proline content was reduced in the JMD group compared to the TC group (*p* < 0.05), and both aspartic acid and serine contents were notably lower in the TC group than in the other groups (*p* < 0.05).

For essential amino acids (EAA), the total content was higher in the TC group than in the FHS group in both muscles (*p* < 0.05). In the LD muscle, valine, methionine, and isoleucine contents were higher in the TC group than in the JMD group (*p* < 0.05), and the lysine content was highest in the JMD group (*p* < 0.05). In the BF muscle, valine, isoleucine, and histidine contents were notably elevated in the TC group compared to the FHS group (*p* < 0.05).

Additionally, the results show that the contents of sweet amino acids in the LD muscle and umami amino acids in the BF muscle were significantly higher in the JMD group than in the TC group (*p* < 0.05). Moreover, bitter amino acids contents were significantly higher in the TC group than in the FHS group (*p* < 0.05).

### 3.5 Principal component analysis

**Figure 1:**
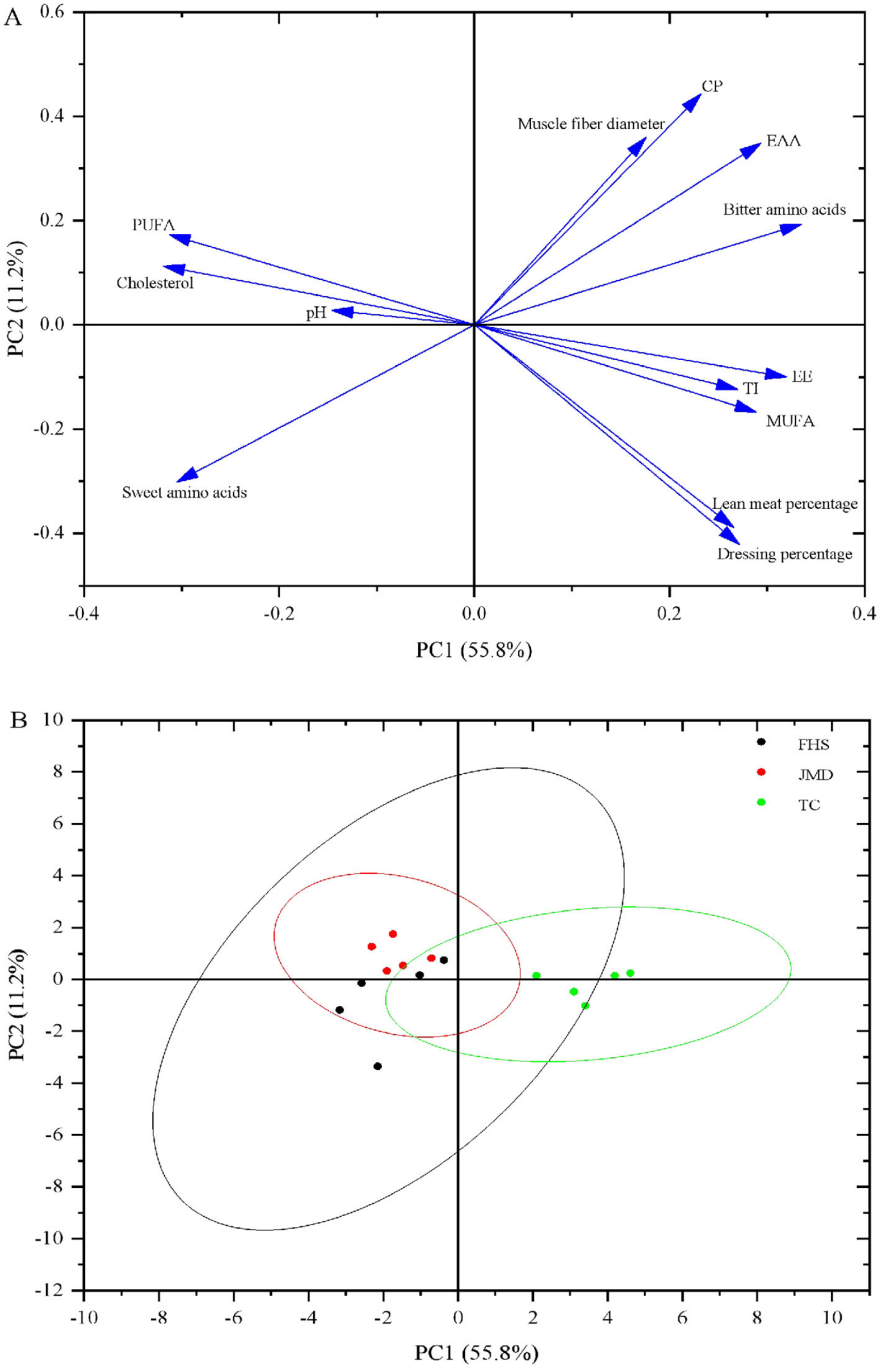
Principal component analysis loading plot and score plot for the major variability variables on the first two PCs were absorbed. **(A)** Loading plot of the investigated variables; **(B)** PC score plot of the first two principal components. CP, crude protein; EE, ether extract; MUFA, monounsaturatedfatty acids; PUFA, polyunsaturated fatty acids; TI, thrombogenic index; EAA, essential amino acids; Bitter amino acids: valine, methionine, isoleucine, leucine, tyrosine, phenylalanine, histidine, argnine; Sweet amino acids: glycine, serine, threonine, alanine, proline, lysine.

Through principal component analysis, Figure 1 shows the overall variation in slaughter performance, nutritional value, and flavor of the meat. PC1 and PC2 explained 67.0% of the variability. Based on the loading values, dressing percentage, EAA, PUFA, sweet amino acids and bitter amino acids were the primary factors for distinguishing the TC group from the FHS and JMD groups (Figure 1A). The TC group was clearly separated from the FHS and JMD groups, while there was an overlap between the FHS and JMD groups (Figure 1B).

## 4 Discussion

### 4.1 Slaughter performance

Cattle production performance is commonly assessed through carcass characteristics, which can be influenced by the feeding system (22). Previous studies have shown that the buffaloes raised on pasture exhibited a lower dressing percentage compared to those raised under intensive management (7). Yuan et al. (12) reported that the dressing percentage and lean meat percentage of indoor-fed yaks at low-altitude were higher than those of grazing yaks. Our findings in this study were consistent with the above results. Additionally, the TC group showed the highest dressing percentage (63.11%), which surpassed the 57.39 and 54.97% observed in the other two groups. Our study indicated that under low-altitude indoor feeding conditions, an improved feeding environment and a more comprehensive nutrient intake contributed to enhanced carcass characteristics in gayals.

Different production system also affected fat deposition. Fat deposition dependents on residual net energy expenditure, with higher dietary energy levels and total carcass fat contents increasing (22), thus indoor feeding led to increased backfat thickness and IMF in the TC group compared to the JMD group (*p* < 0.05), however, no significant difference was found between the FHS and JMD groups (*p* > 0.05). The observed differences may be attributed to several factors, the FHS group was provided with feed of lower nutrient levels relative to the TC group, additionally, due to the brief transition period from grazing to indoor feeding in the FHS group, they had not fully acclimated to the environment of feedlot, moreover, the colder ambient temperature at high altitude required more energy for thermoregulation.

In terms of viscera tissue, in this study, the percentage of lung to slaughter weight increased in FHS and JMD group compared to the TC group. This result may be due to the fact that the lungs are the first organ to respond to hypoxic environments, and in order to adapt to high altitude environments, animals tend to have larger lungs (23). In contrast, the TC group (low-altitude indoor feeding system) faced less hypoxic stress, resulting in a relatively smaller lung proportion.

### 4.2 Meat quality and chemical composition

A diet rich in feed can lead to accumulation of more glycogen in the muscle, the conversion of glycogen to lactate after slaughter contributes to decreasing pH value (24), explaining why TC muscle pH value was significantly lower than that of JMD group (*p* < 0.05). Furthermore, the higher activity of lactate dehydrogenase in the LD muscle of cattle at high altitude (25) resulted in a minimal difference in muscle pH value between JMD and FHS groups, but both were higher than TC group (*p* < 0.05). This study demonstrated that the pH value of the BF muscle was higher than that of the LD muscle, which is consistent with previous findings by Ge et al. (19).

Carvalho et al. (26) suggested that diets rich in lutein and beta-carotene increased the b^*^ value of meat. In our study, the BF muscle b^*^ value in the JMD group was significantly higher than that in the FHS group (*p* < 0.05), indicating a substantial presence of lutein and beta-carotene in the JMD group. The significant increase in BF muscle b^*^ value in the TC group compared to the FHS group (*p* < 0.05) may be attributed to the addition of more corn meal to the TC diet, which, as a feed component may lead to an increase in b^*^ value (27). The change pattern of BF muscle b^*^ values across the three groups was consistent with the trend in fat color reported in Table 2.

Shear force serves as an intuitive indicator of muscle tenderness (28). The size of muscle fiber diameter is a crucial determinant of tenderness, with tenderness diminishing as the fiber diameter increases (29). The LD muscle fiber diameter was larger in the TC group than in the FHS group, and the BF muscle fiber diameter increased in the TC group compared to the FHS and JMD groups (*p* < 0.05). However, IMF also significantly influences meat tenderness, with an increase in IMF leading to a decrease in shear force. In this study, the IMF of the TC group was significantly higher than that of the other two groups (*p* < 0.05). Therefore, the above synthesis explained why no significant difference in shear force was observed among the three groups.

Among the nutrients provided by beef, protein is the most valuable component for consumers. Ge et al. (19) conducted a study to measure the crude protein (CP) content of muscle in nine different breeds of beef cattle in China. The results indicated that there was variability in the CP content within the same breed of cattle across different muscle parts, specifically, the CP content of the LD muscle was the highest in Luxi cattle (23.27%), while the BF muscle of Yunling cattle exhibited the highest CP content (23.66%). Our results showed that the LD muscle of the TC group had the highest CP content (23.57%), followed by the BF muscle of the JMD group (22.45%). Our findings fall within the range of previously reported values, and the variation in CP content may arise from differences in muscle fiber composition and basal metabolic activity between the LD and BF muscles. Additionally, the CP content of the BF muscle was higher in the JMD group than in the TC group (*p* < 0.05), whereas the CP content of the LD muscle did not differ significantly between the TC and JMD groups (*p* > 0.05), consistent with the findings of Yuan et al. (12). This can be attributed to the inverse relationship between moisture content and crude protein in the LD and BF muscles: as moisture content increases, crude protein tends to decrease, and vice versa. Furthermore, no significant difference in moisture content was observed among the three groups in the LD muscle.

Meat serves as a primary dietary source of cholesterol. The dietary guidelines issued by the US Department of Agriculture recommend a daily cholesterol intake of < 300 mg (30). In our study, if 100 g of gayal beef were consumed daily, the cholesterol intake would represent only 7.79%−11.41% of the recommended maximum intake, indicating the potential benefits of consuming gayal beef due to its relatively low cholesterol content. Interestingly, the cholesterol levels in the JMD and FHS groups were significantly higher than those in the TC group (*p* < 0.05), acclimatization to high altitudes showed a significant positive correlation with increasing serum cholesterol, it was mainly due to the fact that cholesterol promotes hypoxic adaptation at high altitude *in vivo* and *in vitro* by inducing the expression of hypoxia-inducible factor (HIF) 1-alpha and inducible nitric oxide synthase (iNOS), thereby protecting hepatocytes against hypoxia-mediated cell death (31).

### 4.3 Fatty acid

Currently, health-conscious consumers are increasingly concerned about the content and composition of fatty acid in beef. A lower AI and TI are utilized to assess the healthy fatty acid composition of meat (7). The h:H ratio is another metric for evaluating meat healthiness, the value above two signifies a healthier fatty acid composition, which is associated with a reduced risk of cardiovascular disease (21). In our study, no statistically significant differences were observed in the AI or h:H ratio among the three groups (*p* > 0.05). This lack of significance may be partly attributed to the relatively small sample size (*n* = 5 per group), which could limit the statistical power to detect subtle differences. Nevertheless, compared with the TC group, the JMD group exhibited a trend toward a lower AI and consistently maintained a higher h:H ratio. More importantly, the TI in the LD muscle was significantly lower in the JMD group than in the TC group (*p* < 0.05), indicating that the meat from gayals in the grazing system had greater health benefits.

C18:3n3 and C18:2n6 are classified as essential fatty acids (EFA). C18:3n3 can be prolongated and desaturated to synthesize C20:5n3, C22:5n3, and C22:6n3 (32). C18:3n3, C20:5n3 and C22:6n3 are EFA that regulate cellular metabolism and function, reduce plasma triglyceride levels, enhance myocardial relaxation and contraction, and mitigate the risk of cardiovascular diseases (33). C18:2n6 is metabolically converted to C20:4n6 through processes of desaturation and chain elongation (34). C20:4n6 affects the activity of ion channels and the fluidity of cell membranes, therefore, it plays a pivotal role in the brain, retina, and central nervous system (35). In our study, C18:2n6, C18:3n3, C20:4n6, C20:5n3, C22:6n3, and C22:5n3 in the LD muscle and C18:3n3, C20:5n3, C22:6n3, and C22:5n3 in the BF muscle of JMD group were significantly higher than those in the TC group (*p* < 0.05), indicating that grazing gayals could provide healthier fatty acid for humans. Interestingly, C20:5n3, C22:6n3, C22:5n3, and C20:4n6 also showed higher levels in the FHS group, which may be attributed to the similar altitude between the FHS and JMD groups and the relatively brief transition period from grazing to indoor feeding for the FHS group.

Grass-fed beef contains a higher concentration of C18:1 t11, which serves as an important precursor for the *de novo* synthesis of conjugated linoleic acid (CAL: C18:2 c9, t11), a potent anti-carcinogenic fatty acid formed within body tissues (36). A linear correlation was observed between dietary C18:1 t11 levels and the rate of CAL synthesis in human subjects (37). In our study, the content of C18:1 t11 in the BF muscle of the JMD group was significantly higher than that in the TC group (*p* < 0.05). Therefore, we can speculate that the BF muscle of the JMD group contains more CAL. Previous research has also confirmed that grass-fed cattle produce beef with a higher content of CLA (38).

The UK Department of Health recommended a PUFA: SFA ratio above 0.45 (39), Gibson et al. (40) reported that n-6:n-3 PUFA ratio close to 1 or 2 and < 5 is optimal for health. In the current study, the PUFA: SFA ratio in the LD muscle of the JMD group was 0.93, which was significantly higher than that of the TC group (*p* < 0.05), and the n-6:n-3 PUFA ratio of LD and BF muscles of the JMD group were 2.82 and 2.92, respectively, which were significantly lower than those in the TC group (*p* < 0.05), these findings further confirmed the potential of the JMD group in balancing fatty acid composition to provide healthier meat products.

### 4.4 Amino acid

Meat serves as a crucial source of high-quality protein, providing humans with a substantial supply of amino acid, some of which play significant physiological roles in human health (41). Methionine and branched-chain amino acids (BCAA) are classified as EAA. In the LD muscle, the levels of methionine, valine, and isoleucine in the TC group were found to be significantly higher than those in the JMD group, while in BF muscle, the levels of valine and isoleucine in the TC group were significantly higher than those in the FHS group (*p* < 0.05), indicating that beef from the TC group was more advantageous for human health in terms of amino acid nutritional value.

Free amino acids are water-soluble compounds that contribute to flavor formation (42), the composition and proportion of amino acid in muscle tissue significantly contribute to the overall flavor profile of the muscle (43). Bitter amino acids, such as valine, methionine, isoleucine, leucine, tyrosine, and phenylalanine, contrast with sweet amino acids, which include glycine, serine, threonine, alanine, proline, and lysine, and umami amino acids consist of aspartic acid and glutamic acid (44). In our study, both the LD and the BF muscles in the TC group exhibited the highest content of EAA, however, the TC group also had the highest bitter amino acids levels, as most EAA were bitter amino acids. Furthermore, the contents of sweet amino acids in the LD muscle and umami amino acids in the BF muscle of the TC group were significantly lower than those in the JMD group (*p* < 0.05), indicating that although the TC group provide high-quality meat enriched in EAA, its poor flavor make the JMD group a more preferred choice for consumers focused on taste. Grazing has been shown to increase flavor amino acid levels in animal muscle tissue, with this elevated content closely associated with natural forage quality and grazing intensity (45). Previous studies have also reported similar findings, showing that grazing increases the contents of sweet and umami amino acids in the LD muscle of Tan lambs compared with the indoor feeding group, an effect that may be attributed to changes in key rumen bacteria, such as *Schwartzia* and *Moryella*, which may affect amino acid deposition in meat (46).

### 4.5 Principal component analysis

Principal component analysis (PCA) effectively visualized the relationships among the three production systems. As shown in the score plot (Figure 1B), the TC group exhibited a distinct separation from the other two groups, whereas significant overlap was observed between FHS and JMD groups. Based on the loading values (Figure 1A), dressing percentage, EAA, PUFA, sweet amino acids and bitter amino acids were the primary factors for distinguishing the TC group from the FHS and JMD groups. Specifically, the TC group displayed a significantly higher dressing percentage and richer content of EAA and bitter amino acids. In contrast, FHS and JMD groups showed elevated levels of PUFA and sweet-tasting amino acids. This result may be attributed to the similarity in altitude between the FHS and JMD groups, as well as the short transition time from grazing to indoor feeding conditions for the FHS group, which resulted in the FHS group appearing closer to the JMD group in terms of slaughter performance, meat quality, and muscle nutritional value.

## 5 Conclusions

This study demonstrates that the production system significantly influences the slaughter performance and meat quality of gayals. Gayals raised under low-altitude indoor feeding conditions (TC group) exhibited superior slaughter performance, including higher dressing percentage, lean meat percentage, and meat: bone ratio, which contributed to an increased yield of beef products. The meat from the TC group was characterized by a lower cholesterol content, higher levels of EAA, and an enriched content of MUFA, particularly C16:1 and C18:1 c9. However, this system also resulted in a higher IMF content and a less favorable fatty acid profile from a cardiovascular health perspective, as evidenced by a higher n-6:n-3 PUFA ratio and a higher TI value. In contrast, gayals subjected to high-altitude grazing (JMD group) displayed reduced slaughter performance, yet produced beef with a healthier fatty acid composition, including higher levels of PUFAs, such as C18:3n3, C20:5n3, C22:5n3, and C22:6n3, a lower n-6:n-3 PUFA ratio, and a lower TI value. This group also exhibited higher contents of flavor amino acids, specifically sweet amino acids in the LD muscle and umami amino acids in the BF muscle, suggesting an enhanced sensory quality. The high-altitude indoor feeding system (FHS group) yielded results intermediate to the TC and JMD groups for most parameters. However, due to the similar altitude and a relatively brief transition period from grazing to indoor feeding, the slaughter performance and meat quality in the FHS group were found to be intermediate between the TC and JMD groups, with characteristics more closely resembling the JMD group.

A major limitation of this study was the restricted sample size (*n* = 5 per group), which was largely constrained by the small population of gayals in China (< 4,000). Future research should aim to incorporate larger sample sizes where feasible and to include longer adaptation periods when transitioning animals between different production systems, to enable a more robust and comprehensive evaluation of how different production systems affect the slaughter performance and meat quality of gayals. Further research could also explore the effects of each production system on animal health, meat sensory attributes, and consumer acceptability, thereby providing foundational data for sustainable production strategies.

## Data Availability

The original contributions presented in the study are included in the article/supplementary material, further inquiries can be directed to the corresponding author.
